# Current practice regarding timing of patent processus vaginalis ligation for idiopathic hydrocele in young boys: a survey of UK surgeons

**DOI:** 10.1007/s00383-017-4085-4

**Published:** 2017-04-19

**Authors:** Matthew Jobson, Nigel J. Hall

**Affiliations:** 1 Department of Paediatric Surgery and Urology, Southampton Children’s Hospital, Southampton, UK; 2 University Surgery Unit, Faculty of Medicine, MP 816, Southampton Children’s Hospital, University of Southampton, Tremona Road, Southampton, SO16 6YD UK

**Keywords:** Hydrocele, Patent processus vaginalis ligation

## Abstract

**Purpose:**

The aim of this study was to determine the current UK practice regarding timing of surgical repair of hydroceles in young boys.

**Methods:**

Through a validated, online survey, participants were asked their preferred management option in five different clinical scenarios across five age ranges.

**Results:**

71 responses were included in the analysis. The most common age to offer surgical intervention for a congenital hydrocele that is stable or increasing in size, or a hydrocele of the cord is 24–36 months. For a stable hydrocele presenting after 12 months of age, the most common age to offer repair is between 36 and 48 months. Approximately ¼ of respondents defer surgery until 4 years of age for any stable hydrocele. For a congenital hydrocele that is decreasing in size, the majority of respondents (57%) do not offer surgical intervention even over 4 years of age. The majority of respondents (61%) do not differentiate between communicating and non-communicating hydroceles when considering age for repair.

**Conclusion:**

These results suggest that there is uncertainty regarding the optimum age for PPV ligation and adequate underlying variability in practice to support a prospective study of the optimum age for hydrocele repair and the natural history of PPV closure.

## Introduction

Idiopathic hydrocele is a common reason for referral to the paediatric surgery outpatient clinic and nearly 3000 operations for hydrocele are performed by surgeons each year in England [1]. Traditional surgical teaching has been that hydroceles that are persistent into the third year of life should be repaired [2]. However, the evidence base for this is unclear, and recently, this surgical dogma has been questioned [1, 3]. Current guidelines offer limited recommendations in relation to the timing of surgery [4, 5]. For example, the International Pediatric Endosurgery Group states that most surgeons advocate observation before 12 months of age and that the majority of patent processus vaginalis’ (PPVs) will close within the first 12–24 months of life; they do not provide a specific recommendation for the age at which surgery should occur [4]. According to national data from the UK, the most frequent age at which surgery takes place is between 24 and 36 months [1].

A recent review of the existing literature in relation to timing of PPV ligation in boys with a hydrocele highlighted a lack of knowledge concerning the natural history of the hydrocele and identified some evidence that hydroceles may continue to resolve beyond the age of 2 years [1]. An improved knowledge of the natural history of this condition may lead to a longer period of observation in anticipation that some hydroceles would resolve spontaneously. Consequently, a period of observation beyond 2 years of age may be justified which could potentially reduce the number of procedures performed (by nearly 900 cases per year in England) [1].

There have been no controlled trials or case series comparing PPV ligation with conservative management of hydroceles presenting at any age. Given the uncertainty regarding the optimal timing for PPV ligation, we believe that further investigation of the natural history of this condition and related surgical practice is justified. The aim of this study was to determine the current practice regarding timing of hydrocele repair amongst UK-based paediatric surgeons and urologists.

## Methods

We designed a web-based survey which was administered using the Research Electronic Data Capture (REDCap) system. REDCap is a secure, web-based application designed to support data capture for research studies, providing (1) an intuitive interface for validated data entry; (2) audit trails for tracking data manipulation and export procedures; (3) automated export procedures for seamless data downloads to common statistical packages; and (4) procedures for importing data from external sources [6].

The survey was piloted within our own department through an iterative process of survey completion and interviews with respondents, to validate individual questions within the survey and the survey overall. The survey was approved by the research committee of the British Association of Paediatric Surgeons (BAPS) and by the secretary of British Association of Paediatric Urologists (BAPU). Formal institutional board approval was not required. Invitations to participate in the survey were distributed electronically to the membership of both these organisations in December 2015. Potential respondents were given approximately 6 weeks to respond and received a reminder about the survey after 3 weeks.

Respondents were asked to indicate their preferred management option for a boy with a hydrocele in five different clinical scenarios across five age ranges. Questions asked in the survey are shown in Appendix 1. A final question asked respondents if they would be interested in participating in a prospective study to better define the natural history of hydroceles. All data were analysed anonymously in Microsoft Excel™ and results are presented descriptively. We decided a priori only to include responses from UK-based Paediatric Surgeons or Urologists practising in a Consultant (Attending) post.

## Results

There were a total of 87 respondents which were limited to 71 for analysis to include only UK-based consultants. The results are summarised in Table 1. There are approximately 190 UK-based paediatric surgery and urology consultants but not all are members of the national organisations through which the survey was distributed. We estimate that respondents to our survey represent approximately 37% of UK consultants.

**Table 1:** Summary of results

		Clinical scenario				
		Hydrocele present since birth, stable in size	Hydrocele present since birth, increasing in size	Hydrocele present since birth, decreasing in size	Hydrocele present after 12 months of age, stable in size	Hydrocele of the cord, stable in size
		Percentage of respondents offering surgical intervention				
Age from which surgical intervention is offered (months)	<12	0	3.2	1.6	N/A	6.6
	12–24	7.6	19	1.6	4.4	4.9
	24–36	34.8	42.9	6.6	24.6	42.6
	36–48	33.3	27	18	34.4	19.7
	>48	24.2	6.3	14.8	29.5	16.4
	Not offered	0	1.6	57.4	6.6	9.8

The most common age to offer surgical intervention for a hydrocele that is present since birth and either stable or increasing in size, or a hydrocele of the cord, is 24–36 months with 36–48 months being the second most frequent age interval for these three clinical scenarios. However, for a hydrocele that has been present since birth but is decreasing in size, the majority of respondents (57%) do not offer surgical intervention, even over 4 years of age.

For a hydrocele presenting after 12 months of age that is stable in size, the most common age to offer repair is between 36 and 48 months, although approximately ¼ of respondents would repair this either earlier or later. Overall, approximately ¼ of respondents defer surgery until after 4 years of age for any hydrocele that is stable in size, regardless of age at presentation.

The majority of respondents (61%) do not differentiate between communicating and non-communicating hydrocele when considering age for repair.

The free text comments section, where respondents were asked to indicate if there is anything else that influences their personal practice, identified two common themes. Namely, a reactive hydrocele associated with a viral illness would prompt a more conservative approach and that very large hydroceles may prompt earlier surgical intervention.

## Discussion

This survey of UK consultant specialist paediatric surgeons and urologists demonstrates variability between surgeons, and across clinical presentation, in the age at which hydrocele repair is offered to young boys. Traditional surgical teaching is that hydroceles that persist into the third year of life should be repaired [2]. UK data would appear to be in keeping with this, since the most frequent age at which surgery takes place between 24 and 36 months [1]. The findings of our survey, however, suggest that over half of specialist children’s surgeons and urologists usually defer surgery until at least 3 years of age for hydroceles that are stable in size, whether congenital or not. Furthermore, for this group of boys, approximately one quarter defer surgery until the age of 4 years. Perhaps unsurprisingly, if there is evidence that the hydrocele is enlarging, a higher proportion of surgeons offer surgery earlier (43% at age 24–36 months and 19% between 12 and 24 months). Conversely, hydroceles that are getting smaller are offered surgery later, or not at all (Table 1). A hydrocele of the cord is a variant that attracts earlier intervention as does a very large hydrocele.

Interestingly, our results differ substantially from the two surveys of the Section on Surgery of the American Academy of Paediatrics that documented practice in North America [7, 8]. The survey in 1993 found that two-thirds of surgeons would offer surgery at the time of diagnosis of a communicating hydrocele, with one-third waiting until between 6 and 12 months of age [7]. This had reduced to 46% in 2003 [8]. Interestingly, just over 40% of surgeons in both surveys perform surgery for non-communicating hydroceles persisting at 1 year of age and only very few surgeons (3% in each survey) deferred surgery for hydroceles until 2 years of age [7, 8]. In the UK, we have shown that upwards of 80% of our respondents (dependent on the clinical situation) would wait until at least 2 years of age before offering PPV ligation. In their review articles, both Lau et al. and Lao et al. recommend surgery for any hydrocele persisting beyond 2 years of age, although the evidence base for this is limited [9, 10].

Unfortunately, the literature documenting the natural history of hydroceles is sparse, presumably since most boys undergo surgery. Koski et al., in a study of 174 patients, concluded not only that an initial period of observation of hydroceles is safe but also that over 60% of boys had resolution of their hydrocele. The mean follow-up period in this study was only 10.8 months [11]. Christensen et al. reported a 76% resolution rate in 39 boys with non-communicating hydroceles that presented at over 1 year of age [12]. They concluded that newly developed hydroceles in boys over a year of age should be watched conservatively for a period of 1 year. In our survey, the majority of respondents (over 95%) wait until at least 2 years of age before offering PPV ligation in this group. Using our study methodology, it is not possible to determine the period of observation employed by an individual surgeon for a hydrocele presenting at a specific time after 1 year of age. The systematic review by Hall et al. suggested that a period of observation of hydroceles beyond 2 years of age may be justified [1] and our results would imply that this is reflected in the current practice of some UK-based paediatric surgeons.

A common viewpoint is that communicating hydroceles should be managed identically to inguinal hernias and should, therefore, all be managed surgically, regardless of the age of presentation [9, 10, 12]. This viewpoint is not reflected in our survey results as only 39% of respondents differentiate between communicating and non-communicating hydroceles.

In their retrospective review, Christensen et al. found that 173 of 178 (97%) of patients with communicating hydroceles underwent surgical intervention [12]. In the five boys who did not undergo PPV ligation, three of them had spontaneous resolution of their hydrocele.

Our study has a number of strengths. The relatively large number of respondents suggests that we have documented current practice in the United Kingdom. We used a validated questionnaire. There are also a number of limitations. Since we did not obtain a response from every UK-based paediatric surgeon or urologist, our sample is only a representation of the current practice in the UK. However, since we have demonstrated variability in the existing sample, we do not believe that a larger sample size will have necessarily altered these results. Since the majority of respondents were specialist paediatric surgeons or urologists, we have not been able to document the practice of adult general surgeons who also operate on children. Finally, since this was a survey-based study, the answers given by participants may not always accurately reflect their actual practice in a specific clinical situation.

## Conclusion

The age at which surgery is offered for hydrocele varies by clinical scenario and age amongst UK-based specialist paediatric surgeons and urologists. Whilst most respondents commonly offered surgical intervention between 2 and 3 years, a significant number of surgeons defer surgery until at least 4 years of age. These results suggest that there is uncertainty regarding the optimum age for PPV ligation and adequate underlying variability in practice to support a prospective study of the optimum age for hydrocele repair and the natural history of PPV closure.

## Appendix 1: UK Paediatric Surgeon/urologist survey on management of hydroceles

Is the primary hospital at which you work based in the United Kingdom?

- Yes
- No

Are you a…?*

- Consultant Paediatric Surgeon
- Consultant Paediatric Urologist
- Trainee
- Other

*If your practice covers both general surgery and urology, please select the option which represents the majority of your work

We will now present you with some short clinical scenarios and ask you to indicate your usual management strategy. For each of the clinical scenarios that follow please indicate your current practice by selecting one of the drop down choices A, B, or C.

A. Discharge without follow-up/re re-referral if persists
B. Offer surgical intervention
C. Arrange outpatient follow-up

1. A boy with a persistent hydrocele has been presented since birth and is stable in size.

a. Seen in clinic at <12 months of age

b. Seen in clinic at 12–24 months of age

c. Seen in clinic at 24–36 months of age

d. Seen in clinic at 36–48 months of age

e. Seen in clinic >48 months of age

2. A boy with a persistent hydrocele has been presented since birth and is increasing in size.

a. Seen in clinic at <12 months of age

b. Seen in clinic at 12–24 months of age

c. Seen in clinic at 24–36 months of age

d. Seen in clinic at 36–48 months of age

e. Seen in clinic >48 months of age

3. A boy with a persistent hydrocele has been presented since birth and is decreasing in size.

a. Seen in clinic at <12 months of age

b. Seen in clinic at 12–24 months of age

c. Seen in clinic at 24–36 months of age

d. Seen in clinic at 36–48 months of age

e. Seen in clinic >48 months of age

4. A boy in whom a hydrocele was first noted at 12 months of age and is stable in size.

a. Seen in clinic at 12–24 months of age

b. Seen in clinic at 24–36 months of age

c. Seen in clinic at 36–48 months of age

d. Seen in clinic >48 months of age

5. Do you recognise a difference between communicating and non-communicating hydroceles or do you treat them all the same?*

*Please assume that a communicating hydrocele is one in which the history or examination findings are suggestive of persistent communication with the peritoneal cavity (i.e., reported fluctuation in size or reducibility on examination), and a non-communicating hydrocele lacks these features.

- Treat them all the same
- Treat communicating and non-communicating differently

6. A boy with a hydrocele of the cord that is stable in size.

a. Seen in clinic at <12 months of age

b. Seen in clinic at 12–24 months of age

c. Seen in clinic at 24–36 months of age

d. Seen in clinic at 36–48 months of age

e. Seen in clinic >48 months of age

Please indicate if there is anything that influences your personal practice of timing of intervention for idiopathic hydroceles that is not otherwise captured on this form (e.g., size of hydrocele—small/moderate/large; history of viral illness, history of pain, presence of tenderness, etc). If so, please state for each whether each would tend to make you operate sooner or later. Please enter brief comments here:

That completes the clinical scenarios. Subject to the outcome of this survey, we are planning a prospective study comparing early and late hydrocele repairs. The study will also be designed to help elucidate the natural history of hydroceles.

Would you be interested in participating in such a study in the future?

- Yes
- No

Would you like to receive a certificate of survey participation (for appraisal/revalidation purposes)?

- Yes
- No
